# A comparison of dynamic cerebral autoregulation across changes in cerebral blood flow velocity for 200 s

**DOI:** 10.3389/fphys.2014.00327

**Published:** 2014-08-26

**Authors:** Martin W.-D. Müller, Mareike Österreich

**Affiliations:** Neurovascular Lab, Department of Neurology and Neurorehabilitation, Kantonsspital LucerneLucerne, Switzerland

**Keywords:** cerebral autoregulation, ultrasound, stroke, transfer function estimates, cerebrovascular physiology

## Abstract

**Objectives**: The dynamic interaction between blood pressure (BP) and cerebral blood flow velocity (CBFV) is not fully understood, especially for CBFV changes lasting longer than 50 s. The interaction between BP and CBFV is relatively well characterized for periods <50 s using transfer function (TF) estimations of phase, gain, and coherence. We used TF estimations to compare the phase and gain for periods >50 s with those for periods <50 s.

**Materials and Methods:** BP and CBFV (of the middle cerebral artery) were simultaneously recorded in 23 healthy subjects (10 men, 13 women, mean age 35 ± 10 years) under normo- and hypocapnia (induced by hyperventilation). TF and coherence estimations were based on Welch's periodogram method with a windowing of 200 s (frequency resolution, 0.005 Hz, corresponding to a period of 200 s). Means of the phase, gain, and coherence were calculated over frequency periods of 0.005–0.02 Hz (sVLF), 0.02–0.07 Hz (VLF), 0.07–0.15 Hz (LF), and 0.15–0.40 Hz (HF) and analyzed using the *t*-test and Pearson correlation.

**Results:** Compared with the VLF range, normo- and hypocapnia phases were slightly but significantly lower in sVLF, while gain and coherence were not different. Hypocapnia induced significant (mostly *p* < 0.01) phase increases and gain decreases as well as coherence decreases in all frequency ranges. The phase and gain correlated significantly (−0.87 < *r* > −0.99) (*p* < 0.001) and inversely in all frequency ranges <0.15 Hz under both respiratory conditions. In some instances, the phase indicated disturbed autoregulation.

**Conclusion:** In the frequency range <0.15 Hz, the phase and gain correlate highly and linearly with high consistency. The phase, gain, and coherence were similar in sVLF and VLF ranges. The phase was slightly lower in the sVLF range than in the VLF range. Notably, the data suggest that autoregulatory failure may occur in healthy persons.

## Introduction

Cerebral autoregulation (CA) describes the brain's ability to maintain a constant blood flow, and hence, a constant metabolic supply despite blood pressure (BP) changes over a wide range. The major response to BP changes is controlled by the pial arteriolar vessels while metabolic stimuli target the intraparenchymal vessels (Kontos et al., 1978; MacKenzie et al., 1979; Kobari et al., 1987). A sudden drop in BP can be used to test vascular responses. This method has been used by Kontos et al. (1978) to describe the response of the pial arterioles in animals and by Tiecks et al. in humans (Tiecks et al., 1995). The resulting vessel response curves were similar in these studies. Tiecks et al. formulated a linear state space model as the first mathematical model to describe the dynamics of this vascular response to a sudden BP drop. Other models for mathematically assessing the dynamics of this interaction between BP and cerebral blood flow velocity (CBFV) (dynamic cerebral autoregulation, dCA) were then developed (Giller, 1990; Zhang et al., 1998; Panerai et al., 1999a,b; Giller and Müller, 2003; Steiner et al., 2003). However, all of the present models have certain shortcomings, indicating a need for further development of the system that transforms BP into CFV.

The most frequently used model for this purpose is a transfer function (TF) approach. Using spontaneous oscillations in BP and CBFV, the TF approach estimates the phase shift (time difference) between the input signal (BP) and the output signal (CBFV) and the gain factor between both signals over a broad frequency spectrum (Zhang et al., 1998; Panerai et al., 1999a). In the TF model, the phase and gain are considered 2 aspects of a high-pass filter that acts primarily in the low frequency range (0.07–0.15 Hz). During normal autoregulation, CBFV usually precedes BP by 40–60° (Müller et al., 2003; Reinhard et al., 2004; Meel-van den Abeelen et al., 2014), indicating that BP is delayed before entering the cerebral circulation. Impaired autoregulation is assumed when the phase is 0° or near 0°. The gain usually increases in the low frequency range, but may decrease with autoregulatory failure. At high frequencies (>0.15 Hz), dCA is less relevant, and CBFV changes associated with heart stroke volume changes are passed through without filtering. However, changes in cerebral blood volume and CBFV also occur at intervals of 100–200 s (Giller, 1990), corresponding to frequencies of 0.01–0.005 Hz. While the TF parameter changes in the very low frequency range (0.02–0.07) are familiar, very little is known about the behavior of the BP/CBFV system (in terms of TF parameters) at frequencies below 0.02 Hz. Increased knowledge of this system's behavior during these long-lasting periods of CBFV changes might explain the difficulties encountered in the simulation of CBFV from BP (Giller and Müller, 2003). A good prediction of CBFV from BP is necessary for using the behavior of the BP/CBFV system to assess the brain's cerebrovascular integrity. The goal of monitoring this integrity is to identify the breakdown of autoregulation sufficiently early to avoid cerebral ischemia in severely ill patients (e.g., head trauma, subarachnoid hemorrhage). The BP/CBFV system has been well characterized with respect to TF estimations in the frequency range of 0.07–0.15 Hz; we thus used this approach to compare the behavior of the system at frequencies <0.02 Hz with those of that at frequencies >0.02 Hz.

## Materials and methods

This study was approved by the local ethics committee.

With their written informed consent, 23 healthy subjects [10 males, 13 females, mean age ± standard deviation (SD), 35 ± 10 years] without cerebrovascular risk factors or neurological diseases underwent recordings of cerebral blood flow velocity (CBFV; with the use of Multi DopX4; DWL; Compumedics Germany, Singen, 2 MHz probe) of the right middle cerebral artery and of BP at the fingertip (Ohmeda 2300 Finapres, Amsterdam, The Netherland) using a TCD device that records these signals simultaneously. The sampling frequency was 100 Hz. The volunteers were lying in a supine position. The Doppler probe was mounted on a light metal TCD probe holder provided by the manufacturer. The middle cerebral artery (MCA) was identified according to the commonly accepted criteria. Using a light face mask, the end-tidal carbon dioxide concentration (EtCO_2_) was measured using the Enhancer 3000sx (Diversified Diagnostic Products, Inc., Houston, Tx). The subjects were allowed to relax in the setting for 5 min before starting the CBFV and BP recordings. For each parameter, the recordings of the baseline and hyperventilation periods were intended to last for a minimum of 9 min. Only recordings without artifacts over the time period of 9 min were included in the analysis. This criterion was fulfilled in 17/23 recordings under baseline conditions and in 18/23 under hyperventilation.

Matlab R2012b (The Mathworks, Inc., Natick, MA, USA) was used for all analyses including statistics. The raw waves were recollected for smoothing at 1 Hz. The new data segments were then normalized to their mean. The coherence and TF estimates of the phase and gain between BP and CBFV were extracted from their respective power autospectra or cross spectra using Welch's averaged periodogram method with a Hanning window length of 200 s, a window overlap of 50%, and a total Fast Fourier Transformation data length of 600 s. The frequency resolution of this setting is approximately 0.005 Hz. For each subject, the coherence, phase (in radians), and gain were calculated over the frequency range of 0.005–0.40 Hz. In 4 frequency ranges, respective means were calculated by averaging the results; we used the frequency ranges of 0.005–0.02 Hz (sVLF, sub very low frequency), 0.02–0.07 Hz (VLF), 0.07–0.15 Hz (low frequency, LF), and 0.15–0.40 Hz (high frequency, HF). Cerebrovascular resistance (CVR) was calculated as BP divided by CBFV and is expressed as the mean from each total recording period. All variables were normally distributed. Results are reported as mean ± SD. Statistical analysis was performed using Pearson correlation analysis and *t*-tests. A *p*-value of <0.05 was considered significant.

## Results

Under baseline conditions, mean BP was 87 ± 6 mmHg and mean EtCO_2_ was 34.0 ± 3.0 mmHg. Under hyperventilation, mean BP remained unchanged (87 ± 8 mmHg), and EtCO_2_ decreased to 22 ± 3.2 mmHg. CVR at baseline was 1.50 ± 0.40 mmHg/cm/s; this value increased to 1.85 ± 0.45 mmHg/cm/s with hyperventilation (*p* = 0.02).

**Figure 1:**
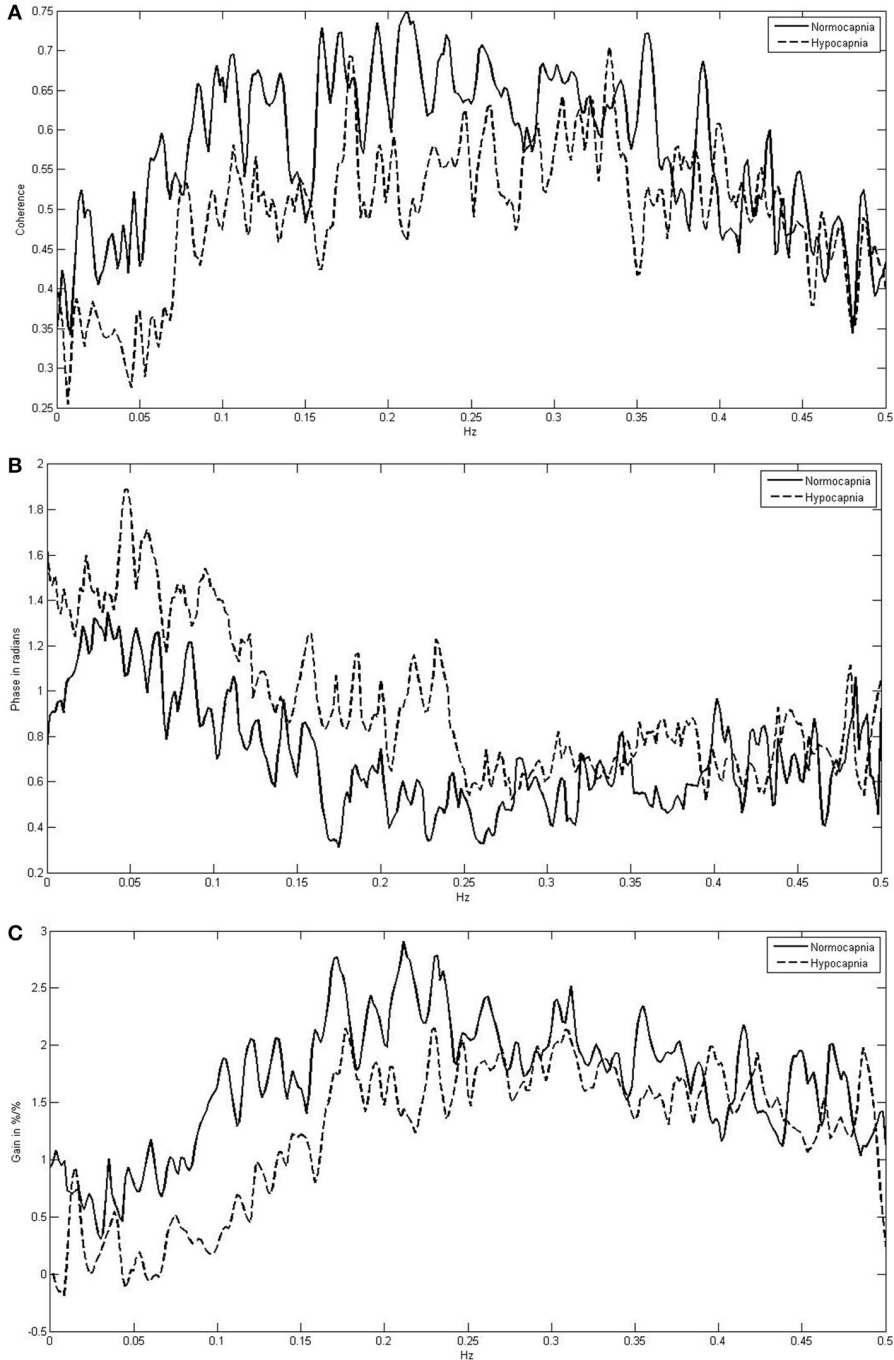
**Frequency-dependent mean coherence (A), phase (B), and gain (C) between blood pressure and cerebral blood flow velocity comparing normocapnia (normoventilation) with hypocapnia (hyperventilation)**.

Figure 1 indicates the means of the coherence, phase, and gain over the entire frequency range (0.005–0.40 Hz) comparing normocapnia with hypocapnia. The observed normocapnic patterns corresponded well to the patterns previously reported when FFT window lengths of 100 s (or less) were used (Giller, 1990; Zhang et al., 1998; Panerai et al., 1999a,b; Müller et al., 2003). The phase was high in VLF and in the initial parts of the LF range; thereafter, the phase decreased steadily toward the HF range. The gain behaved contrary to the phase and was low in SVL; it increased steadily through VLF and LF ranges and was high in HF ranges. The coherence was relatively low in the VLF range and increased toward the HF range. Table 1 summarizes the means for each frequency range and shows that in normocapnia, coherence is equal or above the critical threshold of 0.44 (Gommer et al., 2010). Compared with the VLF range, the phase was slightly but significantly (*p* < 0.01) lower, while the gain and coherence were not significantly different in the sVLF range.

**Table 1:** Comparison of phase, gain, and coherence in different frequency bands with respect to normo- and hypocapnia^*^.

	**Normocapnia**	**Hypocapnia**
**PHASE (IN RADIANS)**		
sVLF	1.01 ± 0.09	1.37 ± 0.08^$^
VLF	1.19 ± 0.10	1.53 ± 0.16^$^
LF	0.87 ± 0.15	1.25 ± 0.18^$^
HF	0.57 ± 0.15	0.96 ± 0.14^$^
**GAIN (IN %)**		
sVLF	0.80 ± 0.17	0.29 ± 0.43^$^
VLF	0.72 ± 0.21	0.13 ± 0.18^$^
LF	1.51 ± 0.37	0.55 ± 0.26^$^
HF	2.25 ± 0.38	1.51 ± 0.33^$^
**COHERENCE**		
sVLF	0.44 ± 0.06	0.33 ± 0.04^$^
VLF	0.48 ± 0.05	034 ± 0.03^$^
LF	0.62 ± 0.05	0.50 ± 0.03^$^
HF	0.65 ± 0.07	0.53 ± 0.05^$^

* *Mean ± SD*.

*For definition of sVLF, VLF, LF, and HF see text*.

$ *p < 0.01*.

Compared with normocapnia, hypocapnia introduced significant changes (Figure 1, Table 1). The phase was significantly higher, while the gain and coherence were significantly lower in all frequency ranges. Only the phase was significantly different (lower; *p* < 0.01) between sVLF and VLF ranges in hypocapnia; the gain and coherence were similar.

**Figure 2:**
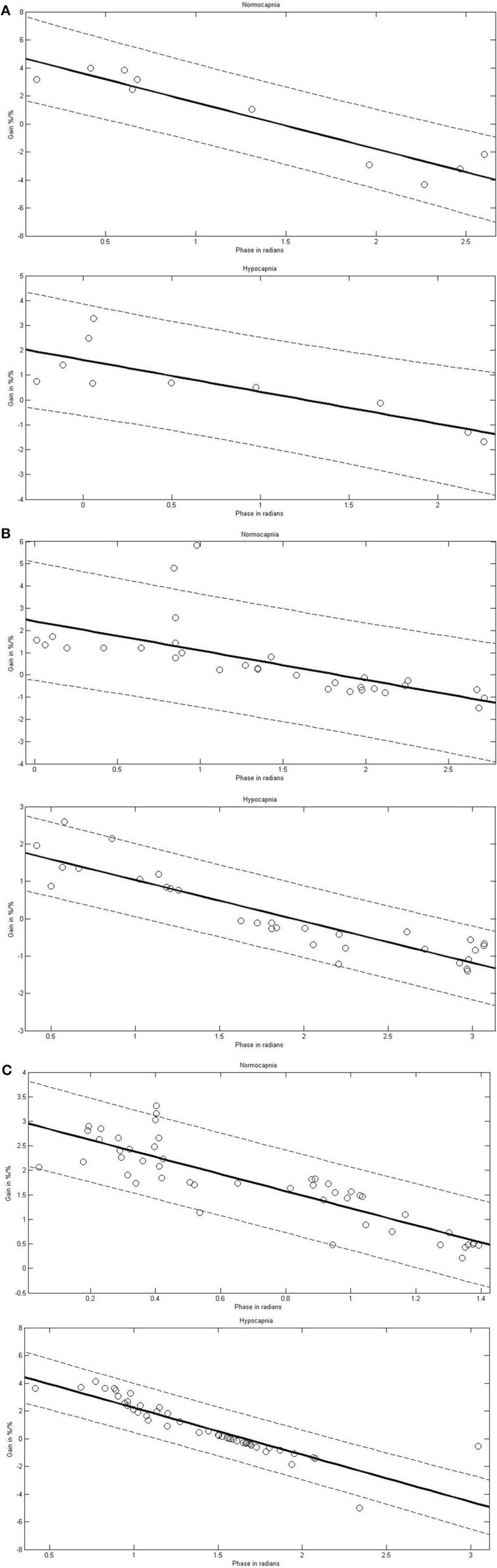
**Linear correlations between phase and gain in different frequency ranges [sub very low frequencies (sVLF; A), very low frequencies (VLF; B), and low frequencies (LF; C)] in normocapnia and hypocapnia**. Correlation line (solid) with 95% confidence interval (dashed line). Each circle indicates a pair of the corresponding means of phase and gain calculated at each frequency step for the investigated subjects; as a result of the different frequency steps, the number of pairs varies.

In the dCA marked phase changes occur predominantly in VLF and LF ranges. Gain changes are also present but less well investigated. When dCA is a filter, investigating the consistency between the phase and gain relationship would be notable. FFT provides a different number of frequency steps in each frequency range. At each frequency step, the mean phase and mean gain for all the subjects were calculated. Using Pearson correlation (Table 2, Figure 2), the mean phase and the mean gain correlated highly significantly among sVLF, VLF, and LF ranges. In the HF range, correlation was high in the normocapnic state, but there was no correlation in the hypocapnic state. It is worthwhile to note that (as shown in Figure 2), phase shift values around 0 radians were present; such low phase values usually indicate a disturbed dCA.

**Table 2:** **Correlation (Pearson correlation coefficients) between phase and gain within different frequency ranges with respect to normo- and hypocapnia**.

	**Normocapnia**	**Hypocapnia**
sVLF	−0.94^$^	−0.82^$^
VLF	−0.65^$^	−0.91^$^
LF	−0.87^$^	−0.88^$^
HF	−0.87^$^	−0.06

$ *p < 0.001*.

## Discussion

Transfer function estimation of dCA revealed that the phase and gain are inversely correlated, with a low gain at high phase shift values and high gains when the phase is low (Giller, 1990; Zhang et al., 1998; Panerai et al., 1999a,b; Müller et al., 2003; Meel-van den Abeelen et al., 2014). Such behavior was determined to be most marked for normocapnia in VLF and HF ranges. Our results agree with this observation, indicating that autoregulation is also present in CBFV changes that occur at periods of 50–200 s.

Dynamic CA has rarely been investigated in periods longer than 50 s. In normocapnia, we found that the phase was slightly but significantly lower in sVFL than VLF. This observation matches those shown in the graphs reported by Giller (Giller, 1990) and Zhang (Zhang et al., 2000). dCA was also investigated in response to changes in CO_2_ and BP. We used hypocapnia as a stimulus and found that the phase increased and the gain decreased over the entire period spectrum (including the sVFL range). The opposite dCA behavior was observed under hypercapnia, (Zhang et al., 1998; Panerai et al., 1999a; Meel-van den Abeelen et al., 2014) with a decreasing phase and an increasing gain in frequency ranges from >0.02–0.15 Hz. There has been no analysis conducted for the sVLF range under hypercapnia. Thus, it might be reasonable to assume from our data that at least a phase decrease is also present in the sVLF range when dCA is impaired. In such a case, dCA behavior in the sVLF range is overall similar to the regulation of faster CBFV changes (at least regarding phase).

In contrast to the phase, the gain did not differ in sVLF and VLF ranges under either normo- or hypocapnia. The gain increased similarly in both frequency ranges when stimulated by hypocapnia. Thus, regarding the gain behavior, CBFV changes occurring between 15 and 200 s are not regulated differently. The gain is considered as an index of vascular tone; interpretation of gain values is not entirely clear because vascular tone should be confounded by CVR, which is inversely related to the gain (Aaslid et al., 1989; Serrador et al., 2005; Zhang et al., 2009). In our subjects, BP did not change between normo- and hypocapnia; thus, increases in CVR should be caused solely by metabolic stimulus. With respect to vascular tone regulation, an increase in CVR led to a decrease in the over all frequency ranges of the gain in our subjects. When BP was increased acutely by infusion of phenylephrine (Zhang et al., 2009) and EtCO_2_ remained unchanged, CVR increased and the gain decreased in the LF range but was unchanged in the VLF range. When BP was decreased by intravenous nitroprusside, the resulting CVR decrease was followed by a gain increase in the LF range (Tzeng et al., 2010) (other frequency bands were not reported). In patients with chronic hypertension, CVR and the gain bewteen VLF and LF ranges correlated inversely (Serrador et al., 2005). Thus, vascular tone is regulated by the inverse relationship between CVR and the gain; this is the most marked relationship in LF and VLF ranges. According to our data, this behavior is similar in the sVLF range.

Over most frequency ranges, the mean gain and mean phase showed a highly stable inverse correlation in our subjects. Remarkably, this strong linearity was observed not only when coherence was high (LF and HF ranges) but also when it was low (sVLF, VLF). The phase ranged from slightly negative to over 2.5 radians. The transfer function model of dCA indicates intact autoregulation when phase shift is positive meaning CBFV precedes BP; an abolished autoregulation is indicated when CBFV follows BP (as indicated by a negative phase shift), or when BP and CBFV oscillate simultaneously (phase shift of 0 radian). Our phase shift results as shown in Figure 2 may indicate that an abolished dCA is present in healthy people. Such disturbances of dCA of healthy people have already been reported twice in reports using linear techniques other than TF estimates (Giller and Müller, 2003; Dineen et al., 2010). The relevance of the coherence should therefore be reconsidered. A strong linear dependence between 2 signals when coherence is low has been demonstrated theoretically (Giller and Müller, 2003), which might indicate a linearity hidden by noise. One suggestion of the origin of noise might be that the actual values of the phase, gain, and CVR are the sum of inverse and contradictory responses to changes in BP and EtCO_2_, and that these changes may differ between vessel types. Whether nonlinear, non-stationary, and/or time-varying approaches can help to overcome these difficulties is a question for future studies (Panerai et al., 1999b; Zhang et al., 2000; Giller and Müller, 2003; Serrador et al., 2005; Mitsis et al., 2006; Czosnyka et al., 2008; Hu et al., 2008; Dineen et al., 2010; Kouchakpour et al., 2010; Marmarelis et al., 2013; Kostoglou et al., 2014; Panerai, 2014).

To summarize, the dCA of CBF velocity changes are similar in periods of 50–200 s and 15–50 s. A small but significant difference in phase shift was observed. In normo- and hypocapnia, there is a strong inverse relationship between the phase and gain at frequencies <0.15 Hz, even when coherence is low. “Autoregulatory failure” appears to be present even in healthy individuals.

## Authorship

Martin W.-D. Müller: study design, statistics and data analysis, data interpretation, manuscript drafting, final approval of the manuscript. Mareike Österreich: study design, data acquisition, data interpretation, revising the manuscript critically for important intellectual content, final approval of the manuscript.

### Conflict of interest statement

The authors declare that the research was conducted in the absence of any commercial or financial relationships that could be construed as a potential conflict of interest.
